# Supplementary material for: In-situ multicore fibre-based pH mapping through obstacles in integrated microfluidic devices
Source: Sci Rep. 2024 Feb 3;14:2839. doi: 10.1038/s41598-024-53106-z (PMC10838297; doi:10.1038/s41598-024-53106-z)
Supplement: Supplementary file 1 — Supplementary Information. [file 41598_2024_53106_MOESM1_ESM.docx]

**Supplementary Information**


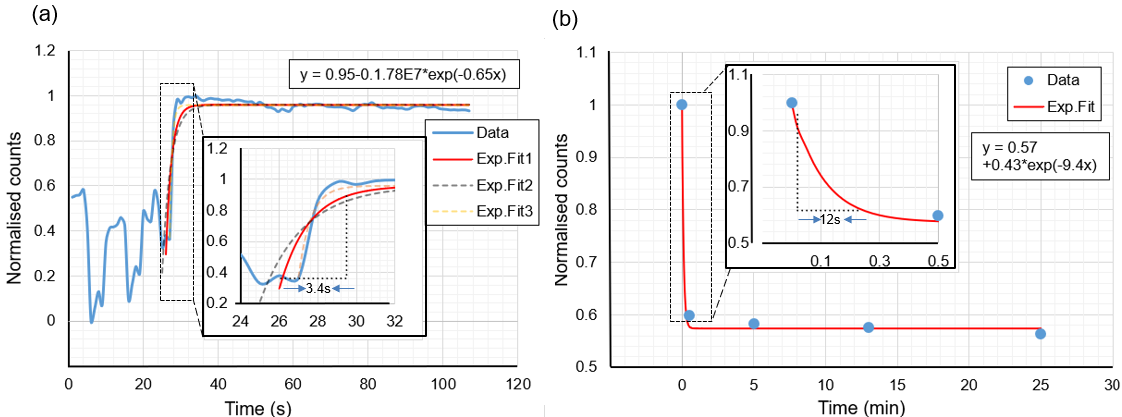


Supplementary Fig S1. Response time of the sensor. (a) Normalised counts of 107 images were recorded on the camera in real-time at 1 frame-per-second. The initial power fluctuation is an artifact of input light source variation. Inset: Depending upon the precise chosen start point for the fitted data, a range of fits can be achieved as shown, indicating a risetime of 3.4 ± 2 seconds. The best fit (Fit 3) achieves an R^2^ value of 0.93. (b) Normalised counts among five intensity profiles were recorded on the camera for pH 2 (first data point) and pH 11 (next four consecutive data points) and the exponential fit to data. Inset: region in which the fall time has been measured from the fit to the data. Based upon the exponential fit, the fall time is estimated to be around 12 seconds. Note that, the data presented in S1 is used to estimate the sensor response so that a much longer waiting time is chosen (300 seconds) for the remaining intensity measurements presented in the paper to ensure that the sensor reading has stabilised.


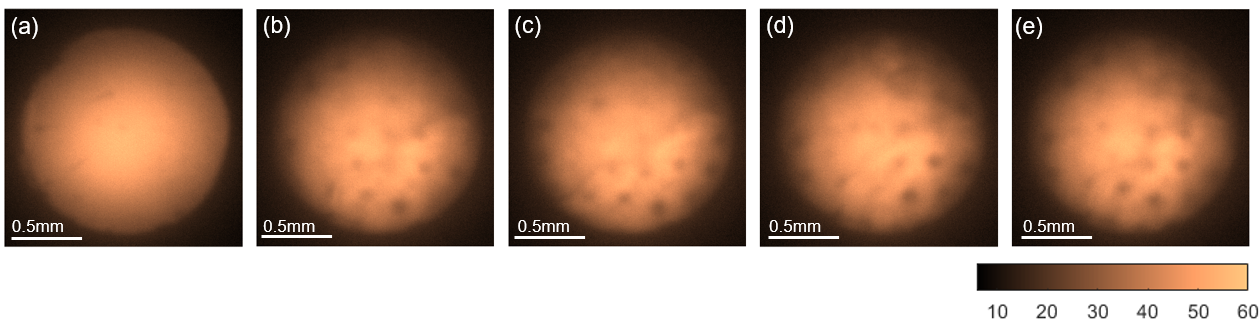


Supplementary Fig S2. False color images of the MCF sensor signal for pH 8.13. (a) Reference image before the obstacles filled in port 2. (b-e) Images taken after inserting the obstacles in port 2. The sensor plug was inserted in port 2 and an image is recorded on the camera. The sensor plug was then removed from the port and reinserted, and the images were taken three more times. Each time, the sensor plug is slightly rotated resulting in changes in the obstacle position across the sensor.


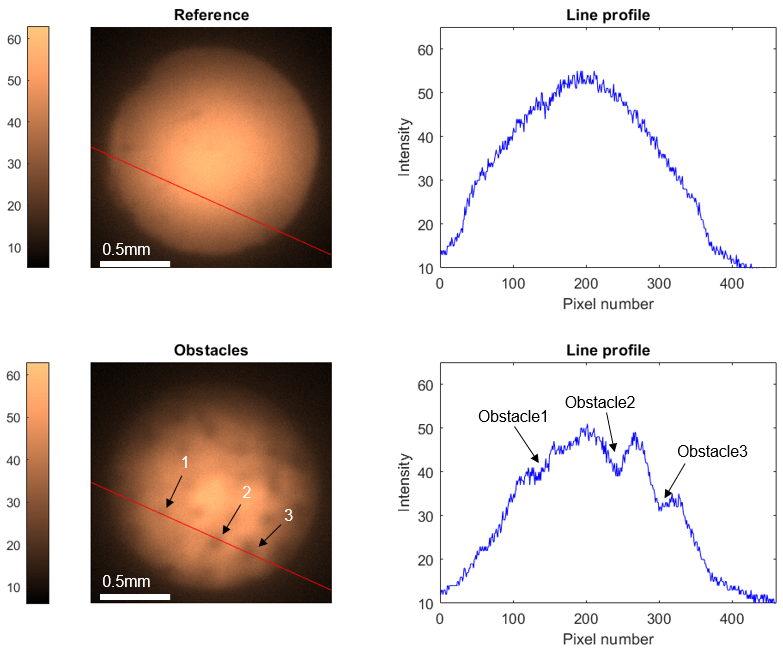


Supplementary Fig S3. Intensity profile across the sensor for the reference (top) and in the presence of obstacles (bottom). The intensity line profile across the sensor when there is no obstacle present shows a clear Gaussian profile while the line profile in the presence of obstacles across the same sensor region (red lines) clearly shows 3 valleys (reduction of signal), indicating the presence of three obstacles (marked as 1,2, and 3 in the bottom left image).
